# Screening of Serum Protein Markers for Avascular Osteonecrosis of Femoral Head Differentially Expressed after Treatment with Yuanshi Shengmai Chenggu Tablets

**DOI:** 10.1155/2018/5692735

**Published:** 2018-03-20

**Authors:** Peng Deng, Jianchun Zeng, Jie Li, Wenjun Feng, Jinlun Chen, Yirong Zeng

**Affiliations:** ^1^The First Clinical Medical College, Guangzhou University of Chinese Medicine, 16 Jichang Road, Baiyun District, Guangzhou, Guangdong Province 510405, China; ^2^The Third Department of Orthopedics, The First Affiliated Hospital of Guangzhou University of Chinese Medicine, Guangzhou, Guangdong Province 510405, China

## Abstract

Avascular necrosis of the femoral head (ANFH) is an a frequently occurring orthopaedic disease with high morbidity. Traditional Chinese Medicine (TCM) Yuanshi Shengmai Chenggu Tablet is a valid prescription for treating steroid-induced femoral head necrosis. However, there are rare investigations about the serum protein marker expression after the acting of drugs on hormone and TCM. In the present study, we aimed to systematically discover and validate the serum biomarkers expression difference in patients with steroid-induced avascular necrosis of femoral head (SANFH) after taking Yuanshi Shengmai Chenggu Tablets (SANFH-TCM), so as to reveal the action mechanism of TCM from the molecular level by using isobaric tags for relative and absolute quantification (iTRAQ) with multiple reaction monitoring quantification. Significant differences in fibrinogen alpha, fibrinogen beta, fibrinogen gamma, fibronectin, C-reactive protein, apolipoprotein A, apolipoprotein D, and apolipoprotein E were found among SANFH, SANFH-TCM, and healthy controls. Therefore, our study proposes potential biomarkers for SANFH diagnosis and for the prognosis of femoral head necrosis after Traditional Chinese Medicine treatment.

## 1. Introduction

Avascular necrosis of the femoral head (ANFH) is a clinical common orthopaedic disease [1–3] with very high morbidity. It is difficult to cure and is one of the major medical problems that have not yet been overcome [4, 5]. Primary osteonecrosis of the femoral head is due to gene or gene mutation of patients. Secondary osteonecrosis of the femoral head could divide into traumatic and nontraumatic osteonecrosis of femoral head [6–8], in which traumatic osteonecrosis of the femoral head is the avascular necrosis of osteocyte caused by interruption of blood flow in the blood vessels in femoral head, which was due to trauma [9, 10]. Its etiology is still unclear. It was demonstrated that long-term and large dosage usage of hormone and drinking are two important factors that cause ANFN [4, 11]. In recent years, with the wide use of corticosteroids clinically, the cases with ANFN have also increased greatly [12, 13]. However, the pathogenesis of steroid-induced ANFN is still unknown. For developing new methods to prevent and treat ANFN, study on the pathogenesis of steroid-induced ANFN is particularly urgent [12, 14, 15].

Recent reports have shown that the occurrence of ANFN could be greatly decreased by an early intervention on high-risk crowds of ANFN who use hormones such as steroid and alcohol [16]. However, the composition of the serum is very complex [17]. It contains high-abundance proteins like albumin and immunoglobulins (mainly IgG), as well as low abundance proteins that are secreted by tissue or cells [18, 19]. Some of them are key proteins involved in signal transduction and regulation [20]. Tan et al. [21] adopted two-dimensional electrophoresis technology and separated 7 differentially expressed proteins between patients with primary femoral head necrosis and normal subjects from 10 pairs serum samples. They found that four important proteins including tissue-type plasminogen activator (t-PA), plasminogen activator inhibitor type 1 (PAI-1), Crosslaps, and anti-p53 antibody were significantly changed and that all of them can be used as the diagnosis serum markers of nontraumatic femoral head necrosis. Although the pathogenesis of ANFN is still unclear and the relevance of this finding with the further clinical application was not reported, analysis of the differentially expressed proteins in the serum could provide useful information.

Traditional Chinese Medicine (TCM) Yuanshi Shengmai Chenggu Tablet is valid and specialty drug for steroid-induced ANFN treatment. Yuanshi Shengmai Chenggu Tablet has obtained the certificate of new medicine in China and has been applied in clinical [22]. Its active ingredients are mainly flavonoids such as vitexin. By clinical studies, it was demonstrated the application of Yuanshi Shengmai Chenggu Tablet can significantly relieve the patients' pain and accelerate the absorption of dead bone and formation of new bone, showing a relatively strong osteogenetic activity [22].

Liu et al. [23] extracted proteins in bone tissue from the femur and humerus bone in rat osteonecrosis model with or without Yuanshi Shengmai Chenggu Tablet TCM treatment and performed proteomics research. They reported that anticoagulating proteins heavy chain II B, phospholipid hydroperoxide glutathione peroxidase, and ubiquitin enzymes E2 (MW: 17 kD) are closely associated with steroid-induced bone necrosis, as well as the therapeutic efficacy of TCM.

In this study we aimed to investigate the differentially expressed protein in serum between steroid-induced ANFN patients with or without TCM treatment (Yuanshi Shengmai Chenggu Tablets). For this purpose, the proteomics method isobaric tags for relative and absolute quantification (iTRAQ) with multiple reaction monitoring (MRM) quantification was adopted in this study, so as to reveal the molecular mechanism of TCM treated the SANFN in the molecular level.

## 2. Material and Methods

### 2.1. Participants

Patients diagnosed as ANFN in the First Affiliated Hospital of Traditional Chinese Medicine University of Guangzhou from February 2014 to February 2015 were included. The ANFN diagnosis was established by referring to standard of adult femoral head necrosis diagnosis expert consensus (2012 edition) and the diagnosis and treatment of avascular necrosis of the expert advice of diagnostic criteria. Patients in active period of ANFN, alcoholics who are simultaneously treated by long-term high dose of glucocorticoids (taken steroid > 10 mg/d longer than 3 years), or patients with combining chronic disease which needs prolonged treatment were excluded in present study. All participants gave written informed consent before being enrolled in the study (AE-2013012011).

### 2.2. Specimens and Groups

Patients with ANFN who have used long-term and high-dosage of steroid (SANFN) were further treated with TCM Yuanshi Shengmai Chenggu Tablet (6 tablets each time, 3 times per day, total 3 months; prepared by the First Affiliated Hospital of Guangzhou University of Traditional Chinese Medicine, Guangzhou, China). Serum samples (*n* = 5) from patients with or without TCM were prospectively collected after obtaining written informed consent. The study protocol was approved by the Ethics Committee of the First Affiliated Hospital of Guangzhou University of Traditional Chinese Medicine. Five healthy subjects were collected during the same period who were sex- and age-matched. Thus, the verification population was divided into 3 groups: steroid-induced avascular necrosis of femoral head (SANFH), SANFH-TCM treatment, and healthy controls.

All serum samples were centrifuged at 1250*g* for 5 min and then 13500*g* for 15 min at 4°C within 1 h of collection. All samples were then stored at −80°C until use.

### 2.3. iTRAQ Analysis of Serum Samples

iTRAQ labeling and mass spectrometry analysis were performed as previously described [24]. Then, six iTRAQ labeled sample polls were generated (steroid-induced avascular necrosis of femoral head, SANFH-TCM treatment, and health controls, each for two subgroups). Briefly, high-abundance serum proteins such as albumin, IgG, and haptoglobin were removed by using the Human 14 Multiple Affinity Removal System (Agilent Technologies, Santa Clara, CA, USA). Then, 50 *μ*g protein of each sample was concentrated and desalted, followed by digestion using trypsin before iTRAQ labeling. Six groups were labeled, including steroid-induced avascular necrosis of femoral head, iTRAQ reagent 113, 116; SANFH-TCM treatment 114, 117; and health controls 115, 118. The six sample groups were mixed, desalted, and dried.

The iTRAQ labeled peptides were separated by Strong Cation Exchange (SCX) chromatography (Bonna-Agela Technologies, Tianjin, China). SCX was carried out on a Polysulfoethyl 4.6 × 100 mm column (5 *μ*m, 200 Å, PolyLC Inc., Maryland, USA). The peptides were eluted at the 45 min gradient from 100% buffer A (10 mM KH_2_PO_4_ pH 3.0, 25% acetonitrile) to 45% buffer B (10 mM KH_2_PO_4_ pH 3.0, 500 mM KCl, 25% acetonitrile) at the flow rate of 800 *μ*L/min on Agilent 1210 LC system. All the fractions were analyzed by MALDI-TOF/TOF 5800 mass spectrometer (AB SCIEX, California, USA). Protein quantification and identification were performed with the Proteome Discoverer (version 1.3, thermos). The default bias correction was used and all quantitative variables were analyzed by the Proteome Discoverer 1.3. Peptide abundances were calculated based on the areas of the monoisotopic peaks. Protein ratios were the average ratios of all quantified peptides. Proteins with quantification *P* value < 0.05 in at least two pairs (113 : 114, 113 : 115, 114 : 115; 116 : 117, 116 : 118, 117 : 118) and with the ratio > 1.2 (the average ratio of two repeat experiments) or ratio < 0.83 were considered as differentially expressed proteins, using a cutoff of 2 times standard deviation [25].

### 2.4. Bioinformatics Analysis

Biomarker candidates were then prioritized using scoring a system based on iTRAQ values from Proteome Discoverer analysis. The cellular component, molecular function, and biological process were analyzed through Gene Ontology (GO) database. The Kyoto Encyclopedia of Genes and Genomes (KEGG) pathway mapping was performed by KEGG Mapper (http://www.genome.jp/kegg/mapper.html), and the enrichment analysis was performed by Blast2GO PRO software (https://www.blast2go.com/, version 2.8).

### 2.5. Validation of Differential Expressed Protein by Multiple Reaction Monitoring (MRM) Quantification

To validate the expression of biomarker candidates, MRM quantifications were performed as previously described [26]. Briefly, 30 *μ*g protein of each sample was digested using trypsin before being desalted. Then, desalted peptide mixtures were loaded onto an Acclaim PePmap C18-reversed phase column (100 Ǻ, Thermo Scientific, Massachusetts, USA) and separated with reversed phase C18 column (300 Ǻ, Bonna-Agela Technologies) mounted on a Dionex ultimate 3000 nano-LC system. Peptides were eluted using a gradient of 5–80% (v/v) acetonitrile in 0.1% formic acid over 45 min at a flow rate of 300 nL/min combined with a Q Exactive mass spectrometer (Thermo Scientific, Massachusetts, USA), and then the eluates were directly entered in Q Exactive MS (Thermo Scientific, Massachusetts, USA), setting in positive ion mode and data-dependent manner with full MS scan within 350–2000 *m*/*z*, full scan resolution at 70000, MS/MS scan resolution at 17500, and MS/MS scan with minimum signal threshold 1*E* + 5, isolation width at 2 Da. To evaluate the performance of this mass spectrometry on the iTRAQ labeled samples, two MS/MS acquisition modes and higher collision energy dissociation (HCD) were employed. And to optimize the MS/MS acquisition efficiency of HCD, normalized collision energy (NCE) was systemically examined, stepped 20%. Each MS/MS spectrum was searched against a mascot database (Uniprot_2015_human database, 20194 protein entries) and a decoy database for FDR analysis (programmed in the software). The search parameters were as follows: sample type, iTRAQ 8-plex (Peptide Labeled); cysteine modification by methyl methane-thiosulfonate; digestion, trypsin enzyme; proteins with, at least, two peptides with a high confidence score (>95%) and a low FDR (estimated local FDR of 5%) were considered positively identified.

### 2.6. Statistical Analysis

All studies to identify biomarkers by iTRAQ/MRM LC-MS/MS were performed on three separate occasions. Statistical analysis was performed using R (version 3.4.2, Bell Laboratories, USA). Analysis of variance (ANOVA) was performed for groups comparison. A *P* value < 0.05 was considered as statistical significantly.

## 3. Results

### 3.1. Populations

A total of 26 patients were included in the present study. Demographic characteristics of present population were summarized in Table 1. All of them were diagnosed as Association Research Circulation Osseous (ARCO) II stage SONFH, and the time windows of being illness were from 6 to 34 months. The mean age was 39.5 years old and 11 (42.3%) of them were males, suggesting that the patients SONFH were younger. Primary cause of 50% of patients was systemic lupus erythematosus, indicating a high risk of long-term high dose of steroid in systemic lupus erythematosus.

### 3.2. Protein Identification and Differentially Abundant Proteins

Serum proteins of steroid-induced ANSF (SANFH) patients, SANFH-TCM treatment patients, and health subjects were screened using iTRAQ. The experiment was repeated twice and detected 399 proteins. Among them, 61 proteins were differentially expressed between SANFH and healthy controls (Table 2), including 35 significantly upregulated proteins (>1.21-fold, *P* < 0.05) and 26 significantly downregulated proteins (<0.83-fold, *P* < 0.05). The top four upregulated proteins in SANFH compared to healthy controls were serum amyloid A-2 (SAA2), Ig lambda, sodium/potassium-transporting ATPase subunit alpha-3 (ATP1A3), and calcium-binding mitochondrial carrier protein Aralar1 (SLC25A12) with fold change values of 4.57, 2.64, 2.07, and 1.95. The top four downregulated proteins were properdin, keratin type I cytoskeletal 9 (KRT9), apolipoprotein (a) (LPA), and tropomyosin alpha-4 (TPM4) with fold change values of −1.77, −1.57, −1.65, and −1.61, respectively.

A total of 74 proteins were differentially expressed between SANFH-TCM and healthy controls (Table 3), including 45 significantly upregulated proteins (>1.21-fold, *P* < 0.05), and 29 significantly downregulated proteins (<0.83-fold, *P* < 0.05). The top four upregulated proteins in SANFH-TCM compared to healthy controls were ATP-binding cassette subfamily B member 9 (ABCB9), fibrinogen alpha, fibrinogen gamma, and fibrinogen beta with fold change values of 17.47, 13.05, 12.67, and 12.11. The top four downregulated proteins were C-reactive protein (CRP), Tubulin alpha-1A (TUBA1A), fibronectin, and LPA with fold change values of −1.73, −1.73, −1.52, and −1.48, respectively.

In addition, a total of 81 proteins were differentially expressed between SANFH-TCM and SANFH (Table 4), including significantly 44 upregulated proteins (>1.21-fold, *P* < 0.05) and 37 significantly downregulated proteins (<0.83-fold, *P* < 0.05). The top four upregulated proteins in SANFH-TCM compared to SANFH were ABCB9, IQ, and AAA domain-containing protein 1 (IQCA1), fibrinogen alpha, and fibrinogen beta, which showed the fold change values of 21.82, 13.86, 13.66, and 13.64, respectively. The top four downregulated proteins were serum amyloid A-2 (SAA2), Ig lambda, CRP, and collagen alpha-1 with fold change values of −2.36, −2.26, −2.24, and −2.04, respectively.

### 3.3. Biomarkers Prediction and Validation

**Figure 1:**
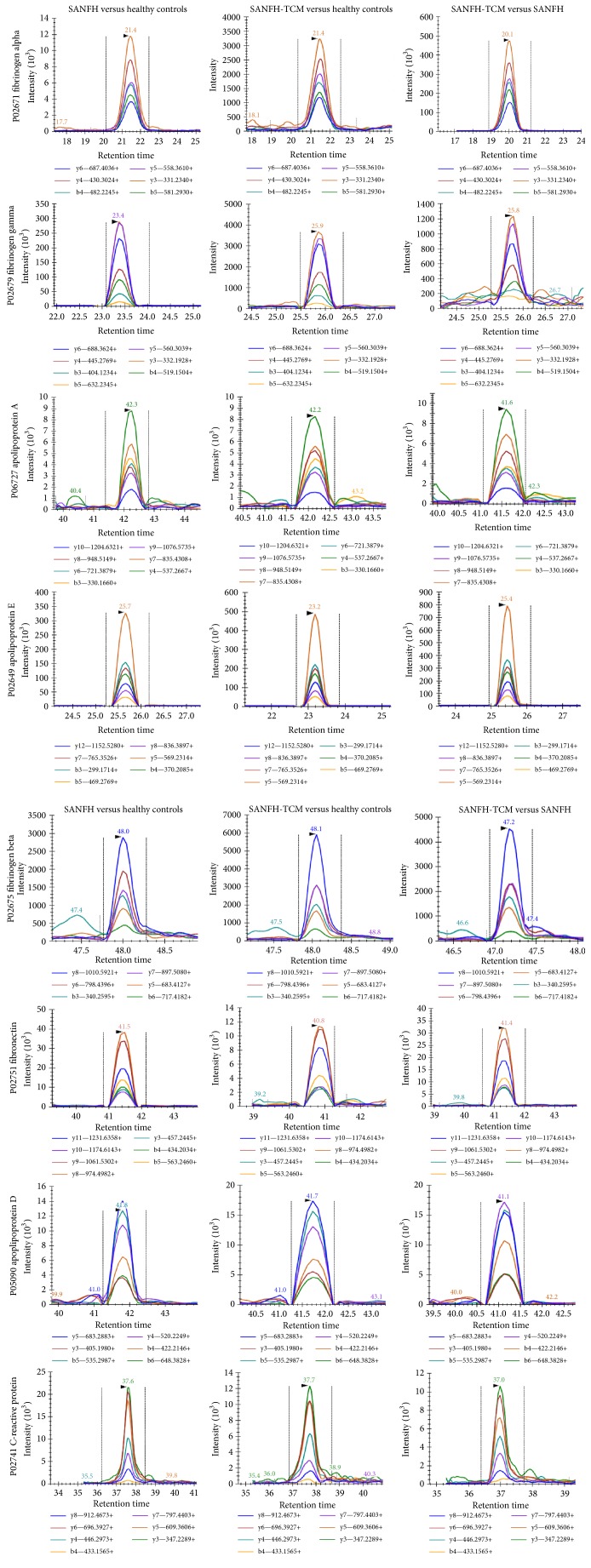
MRM quantification of results obtained from iTRAQ proteomics. MRM was performed to verify the different expressions of selected proteins including fibrinogen alpha, fibrinogen beta, and fibrinogen gamma, fibronectin, apolipoprotein A (LPA), apolipoprotein D (LPD), and apolipoprotein E (LPD), and C-reaction protein in SANFH versus healthy controls, SANFH-TCM versus healthy controls, and SANFH-TCM versus SANFH.

MRM was performed to verify the results obtained from iTRAQ proteomics (Figure 1). The upregulation of fibrinogen alpha, fibrinogen beta, and fibrinogen gamma and apolipoprotein A (LPA) and apolipoprotein D (LPD) in SANFH-TCM versus healthy controls and SANFH-TCM versus SANFH was confirmed by MRM, respectively (*P* < 0.05). Meanwhile, MRM also verified the decreased expression of fibronectin and CRP in SANFH-TCM versus healthy controls and SANFH-TCM versus SANFH identified by iTRAQ, respectively (*P* < 0.05) (Table 5). In SANFH versus healthy controls, CRP and LPA were confirmed to upregulate, and LPD and apolipoprotein E (LPE) were confirmed to downregulate. Using MRM, fibrinogen alpha, fibrinogen beta, and fibrinogen gamma were significantly increased in SANFH compared with healthy controls. However, iTRAQ did not detect significant changes in the expression of fibrinogen alpha, fibrinogen beta, and fibrinogen gamma between SANFH and healthy controls. Although some difference existed between iTRAQ and MRM, all these data added confidence to the results obtained from iTRAQ.

### 3.4. Go Analysis of Differentially Expressed Proteins

The differentially expressed proteins (SANFH versus healthy controls, SANFH-TCM versus healthy controls, and SANFH-TCM versus SANFH) were classified by Gene Ontology (GO) based on their cellular component, molecular function, and biological process.

**Figure 2:**
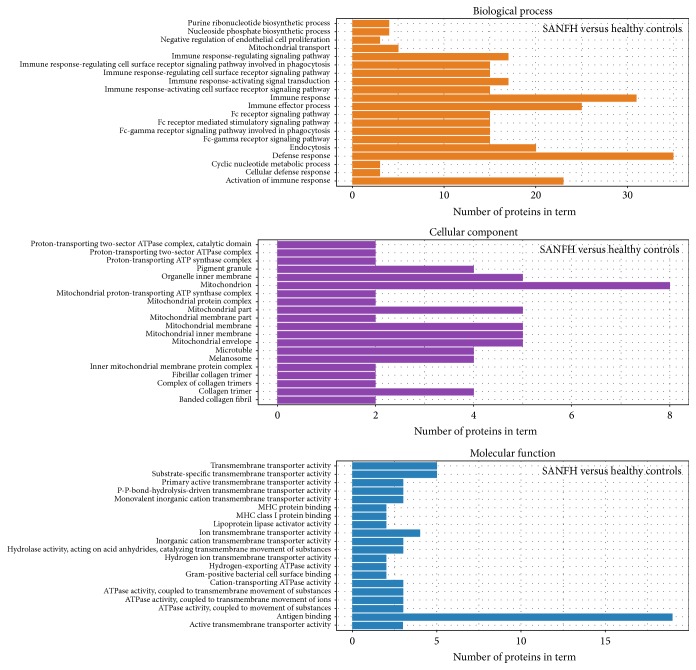
GO analysis of differentially expressed proteins between SANFH and healthy controls. The significantly enriched GO terms concerning biological process, cellular component, and molecular function were shown.

For SANFH versus healthy controls (Figure 2), the top five significantly enriched GO terms concerning biological process were mainly associated with purine ribonucleotide biosynthetic process, nucleoside phosphate biosynthetic process, negative regulation of endothelial cell proliferation, mitochondrial transport and immune response-regulating signaling pathway, and cellular component, in which the top listed five GO terms were proton-transporting two-sector ATPase complex, catalytic domain, proton-transporting two-sector ATPase complex, proton-transporting ATP synthase complex, pigment granule, and organelle inner membrane. With respect to molecular function, transmembrane transporter activity, substrate-specific transmembrane transporter activity, primary active transmembrane transporter activity, p-p-bond-hydrolysis-driven transmembrane transporter activity, and monovalent inorganic cation transmembrane transporter activity were the top five GO terms.

**Figure 3:**
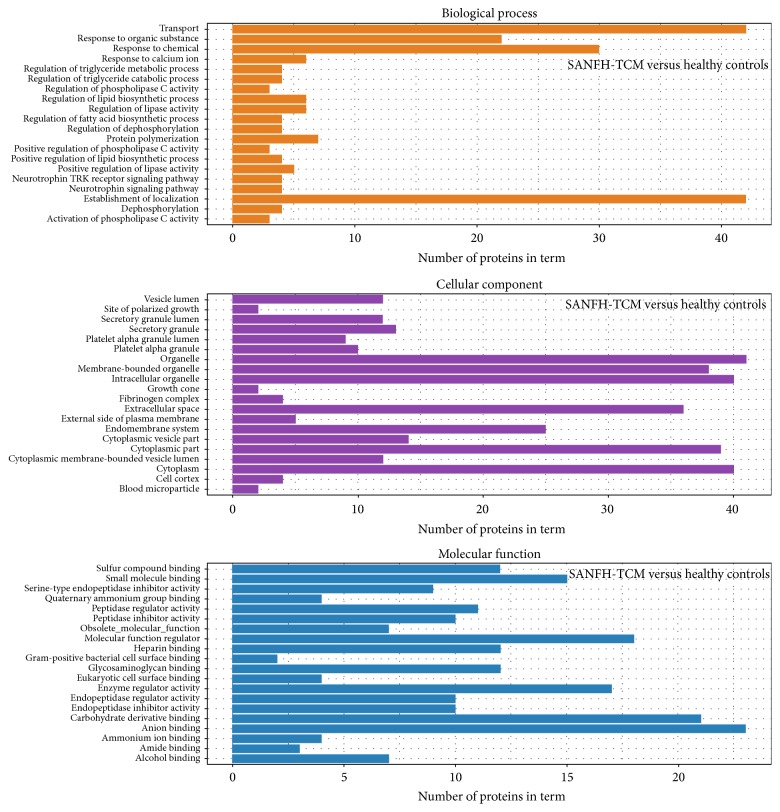
GO analysis of differentially expressed proteins between SANFH-TCM and healthy controls. The significantly enriched GO terms concerning biological process, cellular component and molecular function were shown.

For SANFH-TCM versus healthy controls (Figure 3), transport, response to organic substance, response to chemical, response to calcium ion, and regulation of triglyceride metabolic process were the top five GO terms concerning biological process; vesicle lumen, site of polarized growth, secretory granule lumen, secretory granule, and platelet alpha granule lumen were the top five GO terms concerning cellular component; and sulfur compound binding, small molecule binding, serine-type endopeptidase inhibitor activity, quaternary ammonium group binding, and peptidase regulator activity were the top five GO terms concerning molecular function.

**Figure 4:**
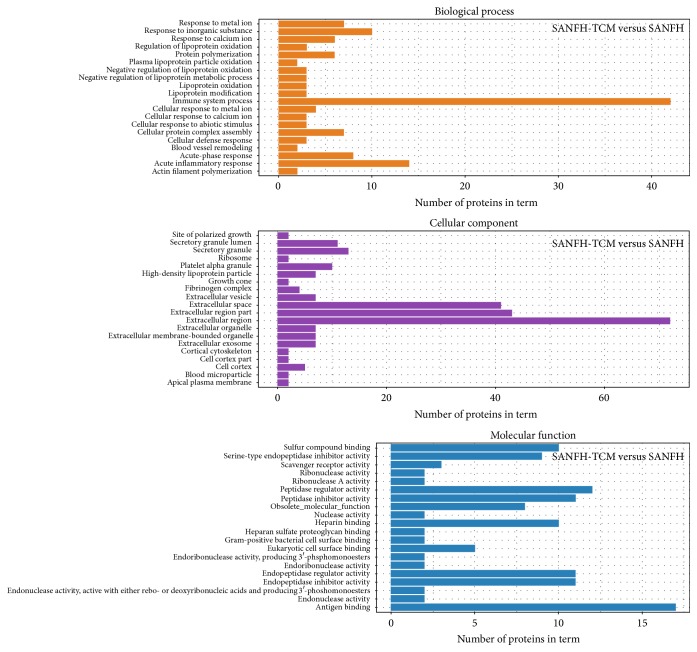
GO analysis of differentially expressed proteins between SANFH-TCM and SANFH. The significantly enriched GO terms concerning biological process, cellular component, and molecular function were shown.

For SANFH-TCM versus SANFH (Figure 4), most enriched GO terms were response to metal ion, response to inorganic substance, response to calcium ion, regulation of lipoprotein oxidation, and protein polymerization in biological process, site of polarized growth, secretory granule lumen, secretory granule, ribosome, and platelet alpha granule in cellular component, and sulfur compound binding, serine-type endopeptidase inhibitor activity, scavenger receptor activity, ribonuclease activity, and ribonuclease A activity in molecular function.

### 3.5. Pathway Enrichment Analysis of Differentially Expressed Proteins

The differentially expressed proteins (SANFH versus healthy controls, SANFH-TCM versus healthy controls, and SANFH-TCM versus SANFH) were mapped to the reference pathways in KEGG database to identify significantly enriched metabolic pathways or signal transduction pathways. In total, 47, 58, and 20 significantly enriched pathways were obtained in SANFH versus healthy controls (Table 6), SANFH-TCM versus healthy controls (Table 7), and SANFH-TCM versus SANFH (Table 8) (*P* < 0.05), respectively. The top listed five pathways were Alzheimer's disease, salivary secretion, Huntington's disease, Parkinson's disease, and oxidative phosphorylation in SANFH versus healthy controls (Table 6); mineral absorption, PPAR signaling pathway, chemokine signaling pathway, adrenergic signaling in cardiomyocytes, and neurotrophin signaling pathway in SANFH-TCM versus healthy controls (Table 7); and chemokine signaling pathway, platelet activation, cytokine-cytokine receptor interaction, glycosylphosphatidylinositol- (GPI-) anchor biosynthesis, and beta-alanine metabolism in SANFH-TCM versus SANFH (Table 8) (*P* < 0.03), respectively. The predicted biomarker LPE was involved in the enriched pathway of Alzheimer's disease in both SANFH versus healthy and SANFH-TCM versus healthy controls. In SANFH-TCM versus healthy controls, LPA was involved in the enriched pathway of PPAR signaling pathway; fibronectin was involved in the enriched pathway of pathways in cancer, small-cell lung cancer, and bacterial invasion of epithelial cells. Fibrinogen alpha, fibrinogen beta, and fibrinogen gamma were involved in the enriched pathway of platelet activation in both SANFH-TCM versus healthy controls and SANFH-TCM versus SANFH. In SANFH-TCM versus SANFH, fibronectin was involved in the enriched pathway of regulation of actin cytoskeleton, and fibrinogen gamma was also involved in the enriched pathway of* Staphylococcus aureus* infection.

## 4. Discussion

This is the first study to reveal proteins associated with steroid-induced avascular necrosis of the femoral head with or without Traditional Chinese Medicine treatment on the proteome level. MRM was used to add confidence to the results obtained by iTRAQ and was attempted for validating 8 proteins (fibrinogen alpha, fibrinogen beta, fibrinogen gamma, fibronectin, C-reactive protein, apolipoprotein A, apolipoprotein D, and apolipoprotein E).

Currently, the pathogenesis theories on femoral head necrosis mainly include [27, 28]: theory of osteoporosis; theory of vascular wall damage or compression and theory of blood lipid disorder [29]; theory of high intraosseous pressure; theory of intravascular coagulation; theory of secondary collision; and so forth [30]. Secondary collision theory [31] considers that osteonecrosis of the femoral head is multifactor disease and it is related to genetic susceptibility factor and exposure to specific risk factors. The occurrence of femoral head necrosis is the collusion result of posterior acquired factors and genetic predisposing factor. Clinical studies also indicate that not all patients that had taken high dose hormone for a long time will suffer from femoral head necrosis and only 10% of patients will be attacked by femoral head necrosis. Though there are many clinical and basis studies about femoral head avascular necrosis, its specific pathophysiological mechanism is still not determined [10, 14]. The beginning of proteomic technology applying in femoral head necrosis is relatively late and there are rare reports. The proteomics study of femoral head necrosis will be helpful to explain the pathological physiology mechanism of femoral head necrosis.

By using meprednisone to induce chicken femoral head necrosis, Li et al. [32] found that there are adipose tissue proliferation and new bone formation through the histological examination; by two-dimensional electrophoresis, 13 protein expression differences were found. Among them, 9 kinds of proteins were downregulated 3 times after hormone treatment, which were serum amyloid P-component precursor, zinc finger protein 28, endothelial zinc finger protein 71, T-box transcription factor 3, cyclin-dependent kinase inhibitor 1, myosin 1D, dimethylaniline monooxygenase, and two kinds of unknown proteins. However, the animal species were different, the cases in the clinical study were few, and the pathogenesis was different, so they lacked comparability and the study results were also different, without representativeness, so they were not sufficient to explain the pathogenesis of femoral head necrosis.

Considering the sampling of bone tissue is an invasive operation, which will bring regional trauma for patients increasing their suffering, the sampling of serum is easier and is also easy for patients to accept. There are few studies on femoral head necrosis. Researchers [3, 33] conducted serum proteomics study on 11 patients with drinking, hormone treatment, or specific femoral head necrosis (3 female and 8 male) and they found 8 protein differential points. Comparing with the serum of healthy volunteers, the serum of patients with femoral head had higher kininogen 1 variant, complement factor C3 precursor, and complement factor H. Besides, patients with femoral head necrosis had significant lower apolipoprotein A-IV precursor, antithrombin III chain B, and gelsolin isoform *α* precursor.

In the present study, we further suggested that the serum amyloid A-2 (SAA2), Ig lambda, sodium/potassium-transporting ATPase subunit alpha-3 (ATP1A3), calcium-binding mitochondrial carrier protein Aralar1 (SLC25A12), properdin, KRT9, LPA, TPM4, ABCB9, fibrinogen alpha, fibrinogen gamma, fibrinogen beta, CRP, TUBA1A, fibronectin, IQCA1, SAA2, and collagen alpha-1 were potential serum marker by iTRAQ and further confirmed the changes of fibrinogen alpha, fibrinogen beta, fibrinogen gamma, fibronectin, C-reactive protein, apolipoprotein A, apolipoprotein D, and apolipoprotein E. The predicted biomarker LPE was involved in the enriched pathway of Alzheimer's disease; LPA was involved in the enriched pathway of PPAR signaling pathway; fibronectin was involved in the enriched pathway of pathways in cancer, small-cell lung cancer, and bacterial invasion of epithelial cells; fibrinogen alpha, fibrinogen beta, and fibrinogen gamma were involved in the enriched pathway of platelet activation; fibronectin was involved in the enriched pathway of regulation of actin cytoskeleton, and fibrinogen gamma was also involved in the enriched pathway of* Staphylococcus aureus* infection. Consistently, it has demonstrated that apolipoprotein A1 is potential risk for femoral head necrosis [34–36]. Fibronectin related to extracellular matrix integrity and adhesion is also an identified serum marker for broiler chickens with femoral head necrosis [35]. Fibrinogen beta was candidate biomarker of infection and inflammation [37] and femoral head necrosis [35]. CRP is an acute-phase protein, negatively correlated with adiponectin level in osteonecrosis of the femoral head [38].

In conclusion, our results identified 74 differentially expressed proteins between SANTH-TCM and healthy controls, 62 proteins between SANFH and healthy controls, and 81 proteins between SANFH-TCM and SANFH. Those upregulated proteins including ABCB9, IQCA1, fibrinogen alpha, and fibrinogen beta and downregulated proteins including serum amyloid A-2 (SAA2), Ig lambda, CRP, and collagen alpha-1 are promising serum diagnosis markers of femoral head necrosis, and also the marker could be used for prognosis of femoral head necrosis after Traditional Chinese Medicine treatment. The key points of treating femoral head necrosis are early diagnosis, early treatment, and reserving femoral head of patients. Our findings on the screening of early serum diagnosis marker of femoral head necrosis are helpful for early intervention on patients with hormone risk factors and preventing femoral head necrosis.

## Figures and Tables

**Table 1:** Demographic characteristics.

In Total *n*	26
Male, *n* (%)	11 (42.3)
Age, yr, mean ± SD	39.5 ± 5.3
History of SONFH, months, median (min, max)	23.1 (6, 34)
Primary disease, *n* (%)	
Systemic lupus erythematosus	13 (50.0)
Anaphylactoid purpura	5 (19.2)
Eczema	4 (15.4)
Psoriasis	2 (7.7)
Thrombocytopenic purpura	1 (3.8)
Fever of unknown origin	1 (3.8)

**Table 2:** Differentially expressed protein between SANFH and healthy controls.

Accession	Description	*P* value	Ratio (mean)
*Upregulated protein*			
P0DJI9	Serum amyloid A-2 protein	0.0000233	4.57402273
P01714	Ig lambda chain V-III region SH	0.000116405	2.64233333
P13637	Sodium/potassium-transporting ATPase subunit alpha-3	0.010160416	2.06683333
O75746	Calcium-binding mitochondrial carrier protein Aralar1	0.008612823	1.95325
P01613	Ig kappa chain V-I region Ni	0.005619256	1.94854167
P01743	Ig heavy chain V-I region HG3	0.00017147	1.93190909
P01742	Ig heavy chain V-I region EU	1.61*E* − 18	1.8255
P0DJI8	Serum amyloid A-1 protein	0.000000019	1.72607353
P01857	Ig gamma-1 chain C region	2.12*E* − 208	1.71611466
P01861	Ig gamma-4 chain C region	0.00000135	1.66788889
P06889	Ig lambda chain V-IV region MOL	0.0000383	1.59966667
P01860	Ig gamma-3 chain C region	6.59*E* − 46	1.59404098
P02741	C-reactive protein	6.38*E* − 146	1.58455808
Q8NBJ4	Golgi membrane protein 1	0.000564362	1.4622
P0C0L4	Complement C4-A	1.33*E* − 10	1.45864286
P12235	ADP/ATP translocase 1	0.016291795	1.4491
P06576	ATP synthase subunit beta, mitochondrial	0.002154497	1.43466667
P13645	Keratin, type I cytoskeletal 10	4.63*E* − 13	1.41348333
P04433	Ig kappa chain V-III region VG (Fragment)	0.000000732	1.412
Q08380	Galectin-3-binding protein	3.33*E* − 45	1.38927444
Q14624	Inter-alpha-trypsin inhibitor heavy chain H4	6.37*E* − 87	1.37619099
P22692	Insulin-like growth factor-binding protein 4	0.025918263	1.362
P01620	Ig kappa chain V-III region SIE	8.22*E* − 09	1.3591875
P02452	Collagen alpha-1(I) chain	1.95*E* − 09	1.3503
O75636	Ficolin-3	2.26*E* − 53	1.341312
P01717	Ig lambda chain V-IV region Hil	0.003213417	1.3341
P14625	Endoplasmin	0.00000523	1.3105
P01621	Ig kappa chain V-III region NG9 (Fragment)	0.000190716	1.2999
P51884	Lumican	0.002279512	1.29618421
P02747	Complement C1q subcomponent subunit C	1.85*E* − 11	1.290775
P25705	ATP synthase subunit alpha, mitochondrial	0.00000765	1.28091667
P22792	Carboxypeptidase N subunit 2	7.1*E* − 09	1.27185227
P09543	2′,3′-Cyclic-nucleotide 3′-phosphodiesterase	0.034218978	1.2625
P18428	Lipopolysaccharide-binding protein	2.71*E* − 47	1.25414706
P01009	Alpha-1-antitrypsin	6.68*E* − 34	1.21782482
*Downregulated protein*			
P01781	Ig heavy chain V-III region GAL	0.00000597	0.82966667
O43852	Calumenin	0.000374747	0.82916667
Q9UK55	Protein Z-dependent protease inhibitor	2.62*E* − 11	0.82908108
O43866	CD5 antigen-like	1.19*E* − 23	0.82744578
Q04756	Hepatocyte growth factor activator	0.000530289	0.821
P63104	14-3-3 protein zeta/delta	0.009022339	0.8194
Q68CQ4	Digestive organ expansion factor homolog	0.000458581	0.81775
P68366	Tubulin alpha-4A chain	0.003314178	0.8143
P04211	Ig lambda chain V region 4A	0.021633191	0.812
Q6Q788	Apolipoprotein A-V	0.000000051	0.8107381
P59665	Neutrophil defensin 1	0.011081692	0.7993
P05164	Myeloperoxidase	0.001604817	0.7984
P02649	Apolipoprotein E	5.01*E* − 267	0.7911827
P04070	Vitamin K-dependent protein C	1.41*E* − 22	0.78128922
Q13103	Secreted phosphoprotein 24	9.56*E* − 09	0.76858333
P02749	beta-2-Glycoprotein 1	0.015081278	0.7567
P01591	Immunoglobulin J chain	2.38*E* − 17	0.75358772
P36980	Complement factor H-related protein 2	0.000968476	0.75325
P04220	Ig mu heavy chain disease protein	0.047631742	0.74433333
P01871	Ig mu chain C region	2.52*E* − 147	0.72098342
Q71U36	Tubulin alpha-1A chain	0.005388449	0.716
P62158	Calmodulin	0.002744078	0.63166667
P67936	Tropomyosin alpha-4 chain	0.002042575	0.62
P08519	Apolipoprotein(a)	1.36*E* − 09	0.606
P35527	Keratin, type I cytoskeletal 9	1.13*E* − 09	0.57923077
P27918	Properdin	2.97*E* − 34	0.57908333

**Table 3:** Differentially expressed protein between SANFH-TCM and healthy controls.

Accession	Description	*P* value	Ratio (mean)
*Upregulated protein*			
Q9NP78	ATP-binding cassette subfamily B member 9	8.47*E* − 09	17.47338
P02671	Fibrinogen alpha chain	0	13.049
P02679	Fibrinogen gamma chain	0	12.66544
P02675	Fibrinogen beta chain	0	12.11164
Q86XH1	IQ and AAA domain-containing protein 1	0.000199	8.9745
P01019	Angiotensinogen	9.49*E* − 09	2.256
Q68CQ4	Digestive organ expansion factor homolog	0.000267	1.882
P03950	Angiogenin	4.8*E* − 10	1.7776
P13637	Sodium/potassium-transporting ATPase subunit alpha-3	0.017893	1.742833
Q8WXD2	Secretogranin-3	1.34*E* − 07	1.71675
P06396	Gelsolin	3.3*E* − 46	1.659898
Q8NBJ4	Golgi membrane protein 1	7.61*E* − 08	1.6227
Q96KN2	Beta-Ala-His dipeptidase	1.18*E* − 06	1.588538
P02775	Platelet basic protein	1.22*E* − 17	1.544688
P06727	Apolipoprotein A-IV	0	1.529316
P01023	Alpha-2-macroglobulin	1.85*E* − 68	1.506488
Q14624	Inter-alpha-trypsin inhibitor heavy chain H4	4.2*E* − 152	1.503549
P02776	Platelet factor 4	1.49*E* − 56	1.487591
Q7Z2Y8	Interferon-induced very large GTPase 1	0.047372	1.4735
P29122	Proprotein convertase subtilisin/kexin type 6	0.002827	1.47275
P05154	Plasma serine protease inhibitor	1.09*E* − 06	1.446654
P14618	Pyruvate kinase PKM	0.005908	1.444167
P06889	Ig lambda chain V-IV region MOL	9.86*E* − 05	1.4295
P01857	Ig gamma-1 chain C region	4*E* − 180	1.427066
P22792	Carboxypeptidase N subunit 2	3.39*E* − 15	1.348591
P01742	Ig heavy chain V-I region EU	3.44*E* − 19	1.343614
P05090	Apolipoprotein D	1.43*E* − 66	1.330211
P51884	Lumican	6.82*E* − 07	1.323868
P01613	Ig kappa chain V-I region Ni	6.17*E* − 05	1.319958
P01860	Ig gamma-3 chain C region	4.37*E* − 19	1.318262
P14625	Endoplasmin	0.000738	1.3125
Q04756	Hepatocyte growth factor activator	4.34*E* − 05	1.312333
P80108	Phosphatidylinositol-glycan-specific phospholipase D	6.28*E* − 16	1.309714
P02787	Serotransferrin	0.03443	1.2721
P00450	Ceruloplasmin	1.43*E* − 27	1.269935
P10720	Platelet factor 4 variant	1.65*E* − 05	1.255714
P80748	Ig lambda chain V-III region LOI	0.003	1.24625
P01009	Alpha-1-antitrypsin	6.2*E* − 51	1.245105
Q9NP79	Coagulation factor XII	0.001987	1.24225
P02671	Retinoic acid receptor responder protein 2	0.008513	1.234583
P02679	Ig kappa chain V-II region TEW	0.024594	1.226038
P02675	Kallistatin	0.016269	1.223857
Q86XH2	Complement C1q subcomponent subunit A	8.72*E* − 05	1.222116
P01020	FERM and PDZ domain-containing protein 1	2.36*E* − 06	1.212885
Q68CQ5	Retinol-binding protein 4	4.81*E* − 08	1.2112
*Downregulated protein*			
Q02818	Nucleobindin-1	0.002012	0.8295
Q9UK55	Protein Z-dependent protease inhibitor	2.62*E* − 11	0.829081
P02656	Apolipoprotein C-III	3.32*E* − 34	0.823015
P62258	14-3-3 protein epsilon	0.04376	0.81975
P01877	Ig alpha-2 chain C region	0.024488	0.817692
Q6Q788	Apolipoprotein A-V	5.25*E* − 08	0.81769
P36980	Complement factor H-related protein 2	0.001599	0.815458
P35542	Serum amyloid A-4 protein	2.65*E* − 29	0.813253
P01620	Ig kappa chain V-III region SIE	1.42*E* − 05	0.800375
O14818	Proteasome subunit alpha type-7	0.024316	0.794833
P18428	Lipopolysaccharide-binding protein	4.26*E* − 75	0.789371
P04438	Ig heavy chain V-II region SESS	0.044412	0.78775
P49721	Proteasome subunit beta type-2	0.026571	0.7765
P04196	Histidine-rich glycoprotein	6.01*E* − 28	0.775008
P02649	Apolipoprotein E	0	0.75853
Q86UD1	Out at first protein homolog	7.29*E* − 08	0.755267
P04211	Ig lambda chain V region 4A	1.19*E* − 05	0.751667
P0DJI9	Serum amyloid A-2 protein	7.71*E* − 05	0.744667
P67936	Tropomyosin alpha-4 chain	0.0005	0.74375
Q8NDV3	Structural maintenance of chromosomes protein 1B	0.000726	0.741833
P62158	Calmodulin	0.005327	0.733667
Q16610	Extracellular matrix protein 1	5.58*E* − 10	0.731882
Q92954	Proteoglycan 4	7.15*E* − 12	0.728857
P59665	Neutrophil defensin 1	0.000151	0.7177
P20742	Pregnancy zone protein	4.88*E* − 71	0.696948
P02741	C-reactive protein	2.7*E* − 173	0.677154
Q71U36	Tubulin alpha-1A chain	0.000833	0.657333
P02751	Fibronectin	0	0.635439
P08519	Apolipoprotein(a)	7.96*E* − 09	0.56335

**Table 4:** Differentially expressed protein between SANFH-TCM and SANFH.

Accession	Description	*P* value	Ratio (mean)
*Upregulated protein*			
Q9NP78	ATP-binding cassette sub-family B member 9	7.38*E* − 10	21.82029412
Q86XH1	IQ and AAA domain-containing protein 1	0.000343733	13.86725
P02671	Fibrinogen alpha chain	0	13.65918665
P02675	Fibrinogen beta chain	0	13.64325401
P02679	Fibrinogen gamma chain	0	13.23321597
P01019	Angiotensinogen	0.000000129	2.391730769
Q68CQ4	Digestive organ expansion factor homolog	0.0000351	2.26525
P03950	Angiogenin	0.00000196	2.0039
P35527	Keratin, type I cytoskeletal 9	3.95*E* − 11	1.928576923
Q8WXD2	Secretogranin-3	0.000728124	1.91025
P27918	Properdin	1.39*E* − 40	1.763869048
P14618	Pyruvate kinase PKM	0.002960903	1.632333333
P05154	Plasma serine protease inhibitor	2.03*E* − 12	1.607923077
Q04756	Hepatocyte growth factor activator	2.81*E* − 08	1.590333333
P02775	Platelet basic protein	2.34*E* − 18	1.56775
P02776	Platelet factor 4	3.43*E* − 84	1.559232955
P02749	Beta-2-glycoprotein 1	0.0000322	1.548
Q96KN2	Beta-Ala-His dipeptidase	0.0000169	1.533115385
P01023	Alpha-2-macroglobulin	1.1*E* − 62	1.519228916
P06727	Apolipoprotein A-IV	0	1.4910721
P06396	Gelsolin	7.08*E* − 33	1.485481481
P01769	Ig heavy chain V-III region GA	0.009729195	1.45675
P10720	Platelet factor 4 variant	0.000000465	1.450190476
P05090	Apolipoprotein D	5.92*E* − 70	1.385849624
P19823	Inter-alpha-trypsin inhibitor heavy chain H2	1.59*E* − 76	1.376936508
P01871	Ig mu chain C region	1.12*E* − 123	1.37344898
P01616	Ig kappa chain V-II region MIL	0.004694041	1.35375
P34096	Ribonuclease 4	0.016807995	1.3485
P29122	Proprotein convertase subtilisin/kexin type 6	0.007174928	1.3275
P80108	Phosphatidylinositol-glycan-specific phospholipase D	1.54*E* − 12	1.320666667
P55056	Apolipoprotein C-IV	0.0000081	1.3169
O95445	Apolipoprotein M	0.00000275	1.314954545
Q9UHG3	Prenylcysteine oxidase 1	0.00000266	1.305045455
P08603	Complement factor H	8.17*E* − 39	1.296578143
P02787	Serotransferrin	0.002337342	1.2947
P05164	Myeloperoxidase	0.020349711	1.282
Q8WWA0	Intelectin-1	0.0000227	1.2735
P29622	Kallistatin	0.0000069	1.267285714
Q9NP79	Immunoglobulin J chain	4.06*E* − 10	1.260973684
Q86XH2	CD5 antigen-like	3*E* − 31	1.248355422
P02671	Plasma kallikrein	6.95*E* − 08	1.236383333
P02675	Serum amyloid P-component	1.03*E* − 24	1.236112319
P02679	Inter-alpha-trypsin inhibitor heavy chain H1	1.7*E* − 30	1.230389423
P01019	Alpha-actinin-4	0.045621384	1.23
*Downregulated protein*			
P04217	Alpha-1B-glycoprotein	0.0000256	0.826346154
P01613	Ig kappa chain V-I region Ni	0.010289928	0.825583333
Q16610	Extracellular matrix protein 1	0.000000945	0.821529412
Q92954	Proteoglycan 4	1.05*E* − 09	0.8145
Q02818	Nucleobindin-1	0.000247875	0.813125
P01742	Ig heavy chain V-I region EU	1.22*E* − 08	0.803428571
P04434	Ig kappa chain V-III region VH (Fragment)	0.030092513	0.802166667
P55774	C-C motif chemokine 18	0.003307656	0.79825
Q9HDC9	Adipocyte plasma membrane-associated protein	0.00000847	0.798083333
P01011	Alpha-1-antichymotrypsin	7.56*E* − 13	0.796217391
P01621	Ig kappa chain V-III region NG9 (Fragment)	0.0000675	0.795
P01717	Ig lambda chain V-IV region Hil	0.0000608	0.78905
P04196	Histidine-rich glycoprotein	2.81*E* − 26	0.783991667
P04433	Ig kappa chain V-III region VG (Fragment)	0.00000651	0.783357143
P35542	Serum amyloid A-4 protein	1.77*E* − 40	0.752563218
P0DJI8	Serum amyloid A-1 protein	4.33*E* − 11	0.748036765
P27824	Calnexin	0.038086684	0.73825
P00740	Coagulation factor IX	6.16*E* − 23	0.733622222
Q9UGM5	Fetuin-B	0.00451302	0.721833333
P0C0L4	Complement C4-A	4.74*E* − 14	0.71725
Q08380	Galectin-3-binding protein	9.69*E* − 59	0.713721805
P04438	Ig heavy chain V-II region SESS	0.039314686	0.70425
P20742	Pregnancy zone protein	9.73*E* − 65	0.698695238
P13645	Keratin, type I cytoskeletal 10	1.17*E* − 19	0.69635
Q86UD1	Out at first protein homolog	3.02*E* − 09	0.694666667
P02751	Fibronectin	0	0.692870958
P04208	Ig lambda chain V-I region WAH	0.000226793	0.6685
P01861	Ig gamma-4 chain C region	2.47*E* − 08	0.655777778
P18428	Lipopolysaccharide-binding protein	5.83*E* − 130	0.640994118
P49721	Proteasome subunit beta type-2	0.019809794	0.6185
P25789	Proteasome subunit alpha type-4	0.00601102	0.609
P01743	Ig heavy chain V-I region HG3	0.0000275	0.607136364
P01620	Ig kappa chain V-III region SIE	1.87*E* − 11	0.5894375
P02452	Collagen alpha-1(I) chain	0.000916641	0.4901
P02741	C-reactive protein	1.19*E* − 243	0.445641414
P01714	Ig lambda chain V-III region SH	1.91*E* − 09	0.442666667
P0DJI9	Serum amyloid A-2 protein	1.16*E* − 08	0.424595238

**Table 5:** MRM was performed to verify the results obtained from iTRAQ proteomics.

Accession number	Description	Relative protein abundance (MRM)			Relative protein abundance (iTRAQ)		
		SANFH versus healthy controls	SANFH-TCM versus healthy controls	SANFH-TCM versus SANFH	SANFH versus healthy controls	SANFH-TCM versus healthy controls	SANFH- TCM versus SANFH
P02671	Fibrinogen alpha	1.8493	62.3799	33.7321	0.95	7.502	7.849
P02675	Fibrinogen beta	3.2104	143.2262	44.6126	0.936	5.8	6.161
P02679	Fibrinogen gamma	1.7341	79.1554	45.6467	0.953	6.721	6.766
P02751	Fibronectin	0.8527	0.3081	0.3614	0.999	0.568	0.571
P02741	C-reactive protein	2.2723	0.522	0.2297	1.559	0.658	0.412
P06727	Apolipoprotein A	1.2723	1.8632	1.4644	1.06	1.532	1.45
P05090	Apolipoprotein D	0.9796	1.4369	1.4668	0.932	1.302	1.341
P02649	Apolipoprotein E	0.6693	0.6865	1.0257	0.759	0.713	0.934

**Table 6:** Differently enriched pathways were obtained in SANFH versus healthy controls.

Pathway_acc	Pathway_Name	*P* value	Protein in Background	Protein in Diff Exp	Protein list
hsa05010	Alzheimer's disease	0.015362	7	4	P25705, *P02649*, P62158, P06576
hsa04970	Salivary secretion	0.032411	5	3	P62158, P04220, P13637,
hsa05016	Huntington's disease	0.057318	6	3	P25705, P12235, P06576,
hsa05012	Parkinson's disease	0.057318	6	3	P25705, P12235, P06576,
hsa00190	Oxidative phosphorylation	0.070274	3	2	P25705, P06576,
hsa04261	Adrenergic signaling in cardiomyocytes	0.088785	7	3	P67936, P62158, P13637,
hsa04915	Estrogen signaling pathway	0.125873	4	2	P62158, P14625,
hsa04020	Calcium signaling pathway	0.125873	4	2	P12235, P62158,
hsa04974	Protein digestion and absorption	0.125873	4	2	P13637, P02452,
hsa04260	Cardiac muscle contraction	0.125873	4	2	P67936, P13637,
hsa04961	Endocrine and other factor-regulated calcium reabsorption	0.162907	1	1	P13637,
hsa04964	Proximal tubule bicarbonate reclamation	0.162907	1	1	P13637,
hsa04960	Aldosterone-regulated sodium reabsorption	0.162907	1	1	P13637,
hsa04070	Phosphatidylinositol signaling system	0.162907	1	1	P62158,
hsa04744	Phototransduction	0.162907	1	1	P62158,
hsa04976	Bile secretion	0.162907	1	1	P13637,
hsa05130	Pathogenic Escherichia coli infection	0.167478	9	3	Q71U36, P63104, P68366,
hsa04918	Thyroid hormone synthesis	0.188181	5	2	P14625, P13637,
hsa04971	Gastric acid secretion	0.188181	5	2	P62158, P13637,
hsa04540	Gap junction	0.253612	6	2	Q71U36, P68366,
hsa04972	Pancreatic secretion	0.299618	2	1	P13637,
hsa04720	Long-term potentiation	0.299618	2	1	P62158,
hsa04911	Insulin secretion	0.299618	2	1	P13637,
hsa05214	Glioma	0.299618	2	1	P62158,
hsa04978	Mineral absorption	0.299618	2	1	P13637,
hsa04973	Carbohydrate digestion and absorption	0.299618	2	1	P13637,
hsa04014	Ras signaling pathway	0.299618	2	1	P62158,
hsa04270	Vascular smooth muscle contraction	0.299618	2	1	P62158,
hsa04910	Insulin signaling pathway	0.299618	2	1	P62158,
hsa05031	Amphetamine addiction	0.299618	2	1	P62158,
hsa04750	Inflammatory mediator regulation of TRP channels	0.299618	2	1	P62158,
hsa04912	GnRH signaling pathway	0.299618	2	1	P62158,
hsa05152	Tuberculosis	0.319543	7	2	P18428, P62158,
hsa05150	Staphylococcus aureus infection	0.334256	18	4	P00751, P0C0L4, P13645, P02747
hsa04120	Ubiquitin mediated proteolysis	0.357215	13	3	P01742, P01781, P01743,
hsa04114	Oocyte meiosis	0.3841	8	2	P63104, P62158,
hsa04919	Thyroid hormone signaling pathway	0.41429	3	1	P13637,
hsa04916	Melanogenesis	0.41429	3	1	P62158,
hsa04064	NF-kappa B signaling pathway	0.41429	3	1	P18428,
hsa04728	Dopaminergic synapse	0.41429	3	1	P62158,
hsa04621	NOD-like receptor signaling pathway	0.41429	3	1	P14625,
hsa05215	Prostate cancer	0.41429	3	1	P14625,
hsa04740	Olfactory transduction	0.41429	3	1	P62158,
hsa04620	Toll-like receptor signaling pathway	0.41429	3	1	P18428,
hsa04713	Circadian entrainment	0.41429	3	1	P62158,
hsa03320	PPAR signaling pathway	0.445992	9	2	P08519, Q6Q788,
hsa05133	Pertussis	0.45325	15	3	P0C0L4, P62158, P02747,

*Note*. Italic font indicated the candidate protein.

**Table 7:** Differently enriched pathways were obtained in SANFH-TCM versus healthy controls.

Pathway_acc	Pathway_Name	*P* value	Protein in background	Proteins in Diff Exp	Protein list
hsa04978	Mineral absorption	0.034949	2	2	P02787, P13637,
hsa03320	PPAR signaling pathway	0.068345	9	4	P02656, P08519, Q6Q788, *P06727*,
hsa04062	Chemokine signaling pathway	0.083136	6	3	P10720, P02776, P02775,
hsa04261	Adrenergic signaling in cardiomyocytes	0.126188	7	3	P62158, P67936, P13637,
hsa04722	Neurotrophin signaling pathway	0.161789	4	2	P62158, P62258,
hsa04915	Estrogen signaling pathway	0.161789	4	2	P62158, P14625,
hsa04260	Cardiac muscle contraction	0.161789	4	2	P67936, P13637,
hsa04114	Oocyte meiosis	0.17539	8	3	P62158, P62258, Q8NDV3,
hsa00410	beta-Alanine metabolism	0.18797	1	1	Q96KN2,
hsa04964	Proximal tubule bicarbonate reclamation	0.18797	1	1	P13637,
hsa00340	Histidine metabolism	0.18797	1	1	Q96KN2,
hsa04070	Phosphatidylinositol signaling system	0.18797	1	1	P62158,
hsa04961	Endocrine and other factor-regulated calcium reabsorption	0.18797	1	1	P13637,
hsa04976	Bile secretion	0.18797	1	1	P13637,
hsa00860	Porphyrin and chlorophyll metabolism	0.18797	1	1	P00450,
hsa00563	Glycosylphosphatidylinositol (GPI)-anchor biosynthesis	0.18797	1	1	P80108,
hsa04960	Aldosterone-regulated sodium reabsorption	0.18797	1	1	P13637,
hsa00230	Purine metabolism	0.18797	1	1	P14618,
hsa04666	Fc gamma R-mediated phagocytosis	0.18797	1	1	P06396,

hsa02010	ABC transporters	0.18797	1	1	Q9NP78,
hsa04744	Phototransduction	0.18797	1	1	P62158,
hsa04611	Platelet activation	0.228912	9	3	*P02675*, *P02679*, *P02671*,
hsa04060	Cytokine-cytokine receptor interaction	0.228912	9	3	P10720, P02776, P02775,
hsa04918	Thyroid hormone synthesis	0.23765	5	2	P14625, P13637,
hsa04971	Gastric acid secretion	0.23765	5	2	P62158, P13637,
hsa04970	Salivary secretion	0.23765	5	2	P62158, P13637,
hsa05200	Pathways in cancer	0.314907	6	2	*P02751*, P14625,
hsa04910	Insulin signaling pathway	0.340991	2	1	P62158,
hsa04912	GnRH signaling pathway	0.340991	2	1	P62158,
hsa04911	Insulin secretion	0.340991	2	1	P13637,
hsa04270	Vascular smooth muscle contraction	0.340991	2	1	P62158,
hsa05214	Glioma	0.340991	2	1	P62158,
hsa04014	Ras signaling pathway	0.340991	2	1	P62158,
hsa04614	Renin-angiotensin system	0.340991	2	1	P01019,
hsa05222	Small cell lung cancer	0.340991	2	1	*P02751*,
hsa05031	Amphetamine addiction	0.340991	2	1	P62158,
hsa04750	Inflammatory mediator regulation of TRP channels	0.340991	2	1	P62158,
hsa04720	Long-term potentiation	0.340991	2	1	P62158,
hsa04973	Carbohydrate digestion and absorption	0.340991	2	1	P13637,
hsa04972	Pancreatic secretion	0.340991	2	1	P13637,
hsa05203	Viral carcinogenesis	0.342177	11	3	P14618, P06396, P62258,
hsa05152	Tuberculosis	0.390394	7	2	P18428, P62158,
hsa04110	Cell cycle	0.390394	7	2	P62258, Q8NDV3,
hsa03050	Proteasome	0.390394	7	2	P49721, O14818,
hsa05010	Alzheimer's disease	0.390394	7	2	P62158, *P02649*,

hsa04740	Olfactory transduction	0.465489	3	1	P62158,
hsa04713	Circadian entrainment	0.465489	3	1	P62158,
hsa04728	Dopaminergic synapse	0.465489	3	1	P62158,
hsa04064	NF-kappa B signaling pathway	0.465489	3	1	P18428,
hsa04930	Type II diabetes mellitus	0.465489	3	1	P14618,
hsa04620	Toll-like receptor signaling pathway	0.465489	3	1	P18428,
hsa04916	Melanogenesis	0.465489	3	1	P62158,
hsa05100	Bacterial invasion of epithelial cells	0.465489	3	1	*P02751*,
hsa00620	Pyruvate metabolism	0.465489	3	1	P14618,
hsa04919	Thyroid hormone signaling pathway	0.465489	3	1	P13637,
hsa04621	NOD-like receptor signaling pathway	0.465489	3	1	P14625,
hsa00330	Arginine and proline metabolism	0.465489	3	1	Q96KN2,
hsa05215	Prostate cancer	0.465489	3	1	P14625,

*Note*. Italic font indicated the candidate protein.

**Table 8:** Differently enriched pathways were obtained in SANFH-TCM versus SANFH.

Pathway_acc	Pathway_Name	*P* value	Protein in background	Proteins in Diff Exp	Protein list
hsa04062	Chemokine signaling pathway	0.01871823	6	4	P10720, P55774, P02776, P02775,
hsa04611	Platelet activation	0.0942118	9	4	*P02675*, *P02679*, *P02671*, P02452,
hsa04060	Cytokine-cytokine receptor interaction	0.0942118	9	4	P10720, P55774, P02776, P02775,
hsa00563	Glycosylphosphatidylinositol (GPI)-anchor biosynthesis	0.2080201	1	1	P80108,
hsa00410	beta-Alanine metabolism	0.2080201	1	1	Q96KN2,
hsa02010	ABC transporters	0.2080201	1	1	Q9NP78,
hsa04666	Fc gamma R-mediated phagocytosis	0.2080201	1	1	P06396,
hsa00900	Terpenoid backbone biosynthesis	0.2080201	1	1	Q9UHG3,
hsa00230	Purine metabolism	0.2080201	1	1	P14618,
hsa00340	Histidine metabolism	0.2080201	1	1	Q96KN2,
hsa04120	Ubiquitin mediated proteolysis	0.2756015	13	4	P01742, P04438, P01769, P01743,
hsa04810	Regulation of actin cytoskeleton	0.2820364	9	3	*P02751*, O43707, P06396,
hsa05150	Staphylococcus aureus infection	0.3119404	18	5	P08603, P0C0L4, *P02679*, P06681, P13645,
hsa05146	Amoebiasis	0.3460206	10	3	*P02751*, O43707, P02452,
hsa05222	Small cell lung cancer	0.3731817	2	1	*P02751*,
hsa04614	Renin-angiotensin system	0.3731817	2	1	P01019,
hsa04978	Mineral absorption	0.3731817	2	1	P02787,
hsa05412	Arrhythmogenic right ventricular cardiomyopathy (ARVC)	0.3731817	2	1	O43707,
hsa05203	Viral carcinogenesis	0.4095525	11	3	P14618, O43707, P06396,
hsa03050	Proteasome	0.4460931	7	2	P49721, P25789,

*Note*. Italic font indicated the candidate protein.
